# Barriers and facilitators to prescribing buprenorphine for treating opioid use disorder among emergency department and other practice setting physicians

**DOI:** 10.3934/publichealth.2025005

**Published:** 2025-01-09

**Authors:** James A. Swartz, Dana Franceschini, Nora M. Marino, Adrienne H. Call, Lisa Rosenberger, Sarah Whitehouse

**Affiliations:** 1 Jane Addams College of Social Work, University of Illinois Chicago, 1040 W. Harrison St., MC (309), Chicago IL 60607, USA; 2 National Opinion Research Center at the University of Chicago, 55 East Monroe St, 30th Floor, Chicago IL 60603, USA

**Keywords:** buprenorphine, medication for opioid use disorders, MAT, emergency department, opioid-related overdoses, Drug Addiction Treatment Act, X-waiver

## Abstract

Despite federal legislation intended to increase the prescribing of buprenorphine as medication for opioid use disorder (MOUD), such as the Drug Addiction Treatment Act (DATA) of 2000, most providers have continued to prescribe to some patients or to not prescribe at all. We aimed to determine the continuing barriers and supports needed for expanding buprenorphine prescribing and compared barriers experienced by emergency department (ED) physicians with those in other practice settings, given the unique aspects of the ED practice setting. We obtained survey data from August through November 2021 from 412 X-waivered Illinois physicians licensed to prescribe buprenorphine as MOUD, 95 (23.1%) of whom worked primarily in a hospital-based ED. Survey questions included: 1) Professional background, practice characteristics, and prescribing practices; 2) barriers to prescribing buprenorphine; 3) barriers to expanding prescribing; and 4) training/additional supports needed to facilitate buprenorphine prescribing. We used bivariate crosstabulations and multivariable OLS and binary logistic regressions to compare the responses of physicians practicing in the ED versus other practice settings and to compare physicians who prescribed buprenorphine in the past year with those who had not. There were few statistically significant differences among the examined subgroups indicating general agreement regardless of practice setting and prescribing status. The most frequently perceived barrier was having an inadequate community-based behavioral health treatment system to which OUD patients could be referred. Insurance reimbursement, difficulties building practice- and community-based systems to support buprenorphine prescribing, and challenges knowing where and how to refer patients for follow-up and ongoing support services were also prominent concerns. Based on study findings, efforts to expand buprenorphine for OUD might focus on providing support to make and manage treatment referrals and expanding the availability of community-based behavioral healthcare services. Building networks of care could potentially have a greater impact on MOUD availability than increasing the number of practitioners trained to prescribe buprenorphine.

## 1. Introduction

Motivated to reduce the increasing number of opioid-related overdoses and fatalities, the U.S. Congress passed the Drug Addiction Treatment Act (DATA) of 2000 [1],[2]. A main objective of DATA was to expand the provision of buprenorphine for treating an opioid use disorder (OUD). DATA granted physicians wanting to prescribe buprenorphine to treat an OUD permission to do so but only after completing an 8-hour training on treating drug addiction and obtaining a license (i.e., known as a “DATA-waiver” or “X-waiver”) from the U.S. Drug Enforcement Administration (DEA) [2]. DATA also limited the number of concurrent patients at 30 to whom a provider could prescribe buprenorphine as medication for OUD (MOUD). At the time, these constraints reflected a compromise between preventing overprescribing and misuse of buprenorphine, while simultaneously broadening treatment access for patients with an OUD [3].

To a significant but limited extent, DATA contributed to an increase in the number of physicians licensed to prescribe buprenorphine. The number of buprenorphine-waivered providers has been estimated as increasing from 17,000 in 2009 to 68,000 in 2018 and between 2016 and 2021, the number of prescriptions written increased by 36% [4],[5]. Moreover, the increases in buprenorphine-waivered prescribers and prescriptions occurred when the number of opioid analgesic prescriptions and prescribers declined. These opposite trends signify that increased buprenorphine prescribing was focused on formulations used to treat OUD and not those prescribed as an analgesic [5].

Despite the increasing number of practitioners prescribing buprenorphine and the number of prescriptions for MOUD post DATA enactment, there were indications significant barriers to prescribing remained. For instance, a recent study of the proportion of persons with an OUD who received any form of MOUD in the past year was only twenty-two percent [6]. Researchers have found that most waivered practitioners under-prescribed relative to their patient limits, with only 10% of prescribers averaging more than 10 patients per month. Moreover, three-quarters of prescribers treated only a few patients before discontinuing prescribing buprenorphine completely [7].

In our research, we found that most waivered Illinois practitioners under-prescribed relative to their patient limit or had not prescribed any buprenorphine as MOUD in the preceding year [8]. Other researchers have identified a similar pattern of buprenorphine under-prescribing relative to DATA-established limits, suggesting that patient caps did not affect MOUD caseloads [9],[10]. Collectively, these studies suggest that most buprenorphine MOUD prescriptions are being written by a minority of high-volume prescribers.

Among the identified persisting barriers were DATA's registration and training requirements [3],[11]. Subsequent legislation reduced and then later removed these requirements at the federal level [12]. In 2006, DATA was amended to increase the patient limit of X-waivered physicians who requested an increase from 30 to 100. And in 2016, buprenorphine prescribing privileges were afforded to nurse practitioners and physician assistants through the Comprehensive Addiction and Recovery Act (CARA, PL. 114–198). In 2018, via the Substance-Use-Disorder Prevention that Promotes Opioid Recovery and Treatment for Patients and Communities Act (SUPPORT, PL. 115–271), physicians with a 100 patient limit for at least a year were permitted to request a limit increase to 275 patients [13]. Further changes occurred in 2021when the U.S. Department of Health and Human Services allowed an exemption to the training requirement for prescribers treating 30 or fewer patients [14]. Section 1262 of the Consolidated Appropriations Act of 2023 (AKA, “Mainstreaming Addiction Treatment” or MAT Act) went further and removed the X-waiver requirement as well as patient limits [15]. As a result of these rule and legislative changes, all U.S. DEA-registered healthcare practitioners with a license to prescribe schedule III opioids and who provided evidence of training in treating drug addiction can prescribe buprenorphine as MOUD, subject to applicable state law [3],[16].

The full extent to which the number of buprenorphine prescribers and prescriptions written have increased post-enactment of the MAT Act remains indeterminant given the recency of the changes as well as mixed findings on how substantial the training requirement and obtaining an X-waiver were barriers to MOUD treatment [12],[17]. For instance, researchers identified the “complexity of the X-waiver process” as an important barrier prior to the MAT ACT whereas other researchers have found that practitioners did not identify the training requirement as a significant impediment to prescribing [18],[19]. Perhaps the strongest evidence that the waiver and training requirements were not the only or even most important barriers to prescribing comes from a recent randomized community-based trial–the Communities that HEAL study–which attempted to increase obtaining an X-waiver and active buprenorphine prescribing through providing training and educational supports to practitioners interested in prescribing buprenorphine as MOUD [20]. The researchers found no difference in either the community rates of practitioners obtaining a waiver or actively prescribing in communities that received the additional training supports compared with those in communities that were part of a wait-list control condition.

These findings and those from other studies support the notion that patient limits and training requirements were not the only or even the major barriers to prescribing buprenorphine for OUD. For instance, a study of licensed U.S. physicians conducted prior to the MAT ACT identified limited education, limited insurance reimbursement, stigma, and the perception of patients with an OUD as “difficult” as MOUD prescribing barriers [21]. Post MAT Act, another remaining barrier has been the patchwork of state laws that continue to require specific credentials and limit practice size much in the same way as DATA [3],[12].

One group of practitioners who might have been less enabled than others by the MAT ACT provisions to prescribe buprenorphine for treating addiction are those whose primary practice is in an emergency department (ED) setting. Although researchers found an increase in the number of ED-related buprenorphine MOUD prescriptions between 2002–2017, other researchers have found the relative increase in number of prescriptions written between 2016 and 2021 to be lower among ED physicians compared to other practice specializations [5],[22].

The ED is a unique practice setting whereby physicians do not typically carry a defined caseload. Although many persons with substance use disorders (SUD) do repeatedly visit the ED for treatment of their SUD and related conditions [23], these visits by definition are not scheduled and those seeking treatment in the ED are not necessarily seen by the same physician(s) who treated them on prior occasions as might be the case if they were receiving care from a primary care physician or addiction specialist. Consequently, MOUD in the ED is more likely than other practice settings to involve buprenorphine induction followed by referral to a community-based MOUD provider rather than induction followed by continued monitoring and ongoing care [24],[25]. The pre-MAT limit on the concurrent number of patients to whom a provider could prescribe was therefore less relevant for ED physicians than other practice groups and unlikely to have been much of a barrier to prescribing MOUD in the ED. On the other hand, there is no reason to believe the X-waiver requirement to obtain SUD training differentially affected ED physicians relative to other practice groups. In fact, ED providers as well as physician practitioners in other specializations have identified the need for more training to facilitate buprenorphine prescribing [17].

Because of the uniqueness of the ED practice setting, there could well be corresponding unique barriers to prescribing buprenorphine post-enactment of the MAT Act. Thus, studies have been conducted to assess the barriers to MOUD prescribing specific to ED-based physicians. For example, Cao et al. reviewed research conducted since 1980 on OUD treatment in the ED [25]. Their enumeration of the reported barriers related to ED-based buprenorphine induction included: Lack of familiarity with prescribing buprenorphine, legal concerns, impact on ED length of stay and patient flow, buprenorphine misuse and diversion by patients, and concerns that providing buprenorphine would increase the volume of patients presenting with an OUD. The lack of community-based providers for referral post-induction in the ED was an additional concern specific to ED physicians located in rural settings. Another recent review of the research on buprenorphine prescribing and barriers to use among ED physicians reached similar conclusions, adding that the requirement of insurance verification and pre-authorization are also barriers in some settings [26].

The Cao et al. findings were similar to those of Hawk and colleagues, who conducted surveys and focus groups with physicians, residents, and advanced practice clinicians working in ED settings [27]. Only a small percentage of Hawk et al.'s sample (3.5%) obtained an X-waiver meaning most lacked experience prescribing buprenorphine as MOUD. Nevertheless, they identified lack of training and experience, difficulty linking to ongoing care, and competing needs for time and resources in the ED as important barriers. To mitigate these barriers, participants indicated a desire for more education and training, treatment protocol development, and feedback on patient experiences to allow for the implementation of MOUD.

Expanding access to buprenorphine in the ED is an important goal in the overall effort to stem opioid-related overdoses and fatalities and increase MOUD treatment engagement not only owing to underutilization but also to the demonstrated effectiveness of ED-based buprenorphine induction [17]. A study of California-based EDs found that nearly fifty percent of patients who initiated buprenorphine treatment while seen in the ED remained engaged in community-based treatment one month post-discharge compared with twenty-three percent who were not provided buprenorphine [28]. Another study of post-treatment engagement following ED-based buprenorphine induction had similarly positive results [29].

The goal of the current study was to expand on the existing body of research that seeks to understand both the barriers as well as facilitators to prescribing buprenorphine as treatment for MOUD in the ED. We believe this is one of the few studies to collect comparative data on these issues from both ED-based as well as non-ED-based physician practice groups. This enabled the determination of which barriers and potential facilitators are specific to ED-based practices and which affect the provision of MOUD treatment more generally. We also wanted to determine if there were unique barriers/facilitators among physicians who actively prescribed buprenorphine as MOUD compared with those who had not actively prescribed in the past year despite having obtained an X-waiver.

## 2. Materials and methods

The University of Illinois Chicago and the National Opinion Research Center (NORC) at the University of Chicago IRBs reviewed and approved study procedures, measures, and survey questionnaires. Participants provided electronic consent for the survey.

### 2.1. Setting

Data for this study were collected as part of a project funded under the SUPPORT Act [30]. Illinois and other states receiving SUPPORT funding were charged with estimating the need for OUD treatment among adult Medicaid beneficiaries and determining the barriers to as well as facilitators for expanding MOUD treatment. In addition to collecting survey data, the SUPPORT project also conducted qualitative interviews with 52 X-waivered healthcare providers, including 10 ED-based physicians, who provided additional information on barriers and facilitators. We used the qualitative interview results in part to structure survey question options as described below. More detail on the methods and findings of the qualitative component of the SUPPORT study as well as other substudies and findings are available elsewhere [8].

### 2.2. Participants

**Figure 1.**
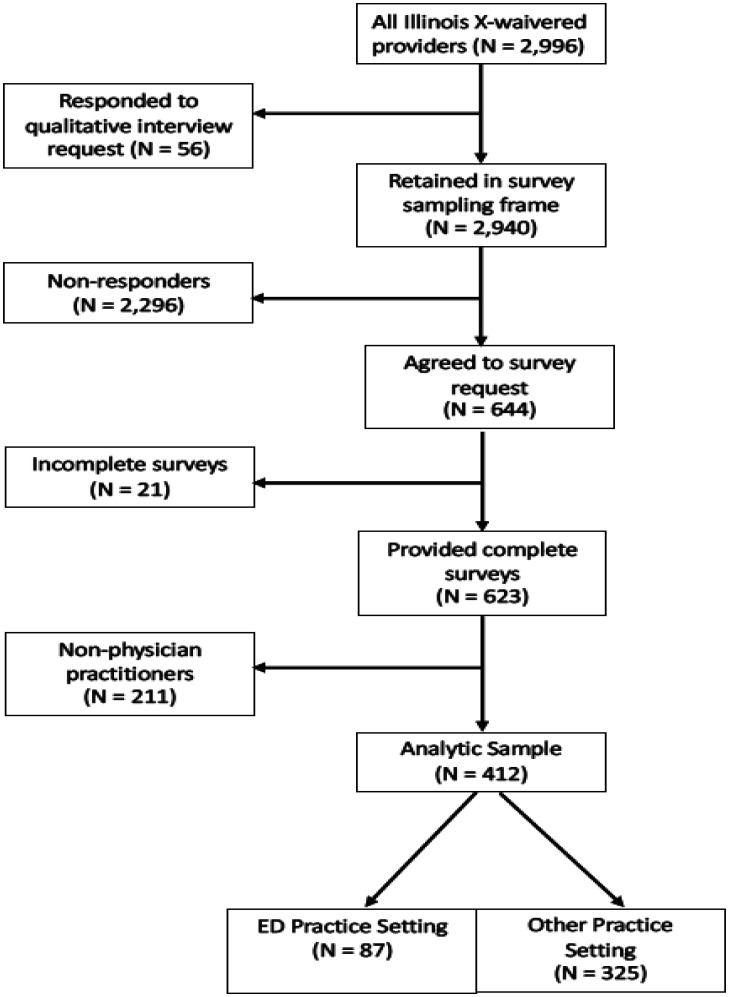
Survey participant recruitment flow chart.

We obtained survey data from X-waivered Illinois healthcare providers licensed to prescribe buprenorphine as MOUD. While the Illinois SUPPORT project was focused on OUD treatment for Medicaid beneficiaries, the surveys were not limited to those providing care to patients insured by Medicaid. We developed the sampling frame based on the complete SAMHSA registry of Illinois X-waivered healthcare providers as of August 2020 (N = 2996). Figure 1 shows a flow chart representing the sample recruitment process and the number of providers included or excluded at each decision point.

We excluded providers who participated in a qualitative interview or who were contacted but refused to complete the interview (N = 56), resulting in a sampling frame of 2940 providers eligible for the survey. We attempted to contact all 2940 providers, receiving 644 responses (21.9%) from which we excluded 21 who did not complete the survey. Of the remaining 623 participants, we excluded 211 non-physician participants (e.g., nurse practitioners, physician assistants), yielding a final analytic sample size of 412; of these, 87 (21.1%) indicated their primary practice was in a hospital-based emergency medicine setting.

### 2.3. Measures

The survey contained questions in the following areas: 1) professional background, practice characteristics, and prescribing practices; 2) barriers to prescribing buprenorphine faced by providers; 3) barriers to expanding prescribing; 4) specific trainings needed to facilitate buprenorphine prescribing; and 5) supports needed to increase buprenorphine prescribing. In constructing the questions on barriers and how best to reduce barriers and increase needed supports, we used information provided by the qualitative interviews to populate question options. For example, if multiple interviewed providers identified a specific barrier, it was included as a response option for the corresponding survey question. A copy of the survey questionnaire is provided as supplemental material.

#### 2.3.1. Professional background and practice setting

Questions on background and practice settings included practice zip code, which we used to categorize each participant by state and whether they practiced in a rural or urban setting; current X-waiver patient limit; whether the provider prescribed buprenorphine in the past year or month; percentage of their practice composed of MOUD patients; and MOUD patient ages, race/ethnicity, and whether the providers saw any underserved populations such as those experiencing homelessness, those with criminal justice involvement, undocumented immigrants, or persons identifying as LGBTQ+.

#### 2.3.2. Barriers experienced prescribing buprenorphine

To assess barriers to administering buprenorphine, participants were asked to indicate their level of agreement with a list of possible barriers. The list included statements such as: Providers who offer MOUD services are stigmatized; [There are] insufficient MOUD providers in the community; [There are] insufficient behavioral health treatment referral resources to support my patients who receive MOUD services, etc. Responses were captured using a 5-point Likert-like scale that ranged from 1 (strongly disagree) to 5 (strongly agree).

#### 2.3.3. Barriers preventing buprenorphine prescribing

Providers who had not prescribed buprenorphine in the past year were asked the following question: “Which of the following reasons prevent you now, or prevented you in the past from prescribing buprenorphine for treating OUD?” Participants could then select from among 10 response options that included: “Insufficient education/background in providing care for people with OUD; insufficient support from my employer; and limited demand for services, etc. Selected options were scored as Yes (1) and non-selected options as No (0).

#### 2.3.4. Trainings needed

As prior studies of buprenorphine prescribing for MOUD have identified additional training as a commonly identified need, we asked participants what specific MOUD training topics would be helpful to their practice. Participants could select from a list of 9 training topics such as skill building and practice in prescribing; medication dosing; prescribing to patients with comorbidities; and detox regimen before administering buprenorphine, etc. Selected options were scored as Yes (1) and those unselected as No (0).

#### 2.3.5. Additional support needed

We presented providers with a list of 7 potential supports and asked them to select which would help increase buprenorphine prescribing. Response options included: Administrative support; case management support; assistance navigating referrals; and a support network of other prescribers and mentors, etc. Selected options were scored as Yes (1) and unselected options as No (0).

### 2.4. Procedures

Preliminary versions of the survey were sent to content experts for review and suggested edits or additions after which revisions were made to improve flow and question-wording. The final set of survey questions was programmed in Voxco, a secure, web-based software platform designed to support data collection for clinical research studies. Each provider was recruited for the survey with up to two mailed letters and several reminder emails. The recruitment letters contained a link by which the survey could be accessed with informed consent collected electronically prior to the start of the survey. We collected survey data from August through November 2021. Providers who completed the survey were compensated for their time with a $50 gift card.

### 2.5. Analyses

We analyzed weighted survey data using Stata v.17.1 [31]. Weighting of the survey data was performed using a two-step process implemented to adjust for survey non-response and calibration to the total in-scope population. Weights were calculated using four weighting variables: Region (Chicago metro or greater Illinois), provider type (e.g., physician, nurse practitioner, physician's assistant), registry status (X-waiver status is publicly viewable or not available to the public), and geography (rural, suburban, urban).

We first generated a series of bivariate statistics to check for missing data or out-of-range responses. Given that data entry rules and skip patterns were enforced by the Voxco software, these issues were minimal and required no corrective actions; no cases were discarded or modified owing to invalid or missing data. We then ran bivariate descriptive statistics for the sample, disaggregated by practice setting (ED vs. Other) comparing professional backgrounds, practice characteristics, and prescribing practices. Statistical significance was assessed on the weighted survey data using design-based F-tests.

For the multivariable analyses of binary responses, we ran logistic regression models and for the analyses of responses captured using a Likert-like scales, we used ordinary least squares regressions. All models used the weighted data and estimated effects for practice setting (i.e., ED versus other), prescribing status (i.e., did or did not prescribe buprenorphine as MOUD in the past year), and the interaction between setting and prescribing status. The models assessing MOUD barriers and supports, were run using the full sample (N = 412). We restricted the analyses to those who reported they had not actively prescribed buprenorphine in the past year (N = 115) for responses related to non-prescribing. Because of the large number of statistical comparisons and the potential for an inflated type-I error rate, we report results as significant only for those where p < 0.01 [32].

## 3. Results

### 3.1. Descriptive statistics

Table 1 shows the professional backgrounds, practice characteristics, and buprenorphine prescribing by practice setting. The majority of surveyed providers (77.7%) indicated their practices were located in the Chicago region and in an urban (57.8%) or suburban (37.0%) location. Just over forty-five percent said their X-waiver patient limit was at the minimum of 30 concurrent patients with higher proportions of inactive prescribers in both the ED (59.9%) and in other practice settings (58.6%), indicating they had a 30-patient limit as compared with active prescribers working in the ED (47.4%) or other practice settings (38.6%). Regardless of prescribing status, a surprisingly high percentage of the physicians surveyed (21.3%) did not know their X-waiver limit, with the highest level of uncertainty (33.8%) expressed by inactive prescribers in practice settings other than the ED.

A significantly lower percentage of ED-based physicians who had prescribed buprenorphine in the past year (43.3%) said they had prescribed in the past month compared with physicians in other practice settings (79.1%). Regardless of practice setting, the majority of surveyed providers (84.8%) said that MOUD patients constituted about 0% to 25% of their current caseloads. Only a relatively small percentage of providers (4.8%) prescribed to adolescents between the ages of 13 to 17 years of age with almost all (98.7%) indicating they prescribed to adults between the ages of 25 to 64 years old. ED-based physicians were more likely to say they prescribed to racial/ethnic minorities such as American Indian or Alaska Native, Asian, or Native Hawaiian or Pacific Islander compared with physicians in other practice settings. There were no statistically significant differences by practice setting for other MOUD patient populations such as persons who are homeless, those involved in the criminal justice system, undocumented immigrants, or those identifying as a sexual or gender minority.

### 3.2. Buprenorphine prescribing barriers

As shown in Table 2, there were relatively few significant differences by either practice setting or prescribing status in terms of provider barriers to prescribing buprenorphine as MOUD. Across both provider types and practice settings, three of the five barriers with the highest levels of endorsement pertained to insurance coverage and reimbursement with the remaining two having to do with the lack of referral networks and treatment resources. Ordered from highest to lowest, the most strongly endorsed prescribing barriers were: insufficient behavioral health treatment referral sources (M = 3.9, SE = 0.05; insufficient community-based MOUD treatment providers (M = 3.6, SE = 0.05); delay in receiving insurance reimbursement (M = 3.6, SE = 0.05); lack of knowledge about insurance coverage (M = 3.4, SE = 0.05); and insurance does not provide sufficient reimbursement to cover the cost of providing MOUD services (M = 3.2, SE = 0.07).

Among the few statistically significant differences found, compared with providers in other practice settings providers in an ED-based setting had a lower endorsement score for the barrier related to having too large a caseload to see new MOUD patients (b = −0.09, p < 0.001). Compared to inactive prescribers, active buprenorphine prescribers also had lower endorsement scores (b = −0.07, p < 0.001) for this same item. Additionally, there were two significant differences between active and inactive prescribers regardless of practice setting, with active prescribers having lower scores for the barrier pertaining to their practice not supporting MOUD providers (b = −0.94, p < 0.001) as well as for their current caseload being too large to see new MOUD patients (b = −0.7, p < 0.001). No other regression estimates yielded statistically significant differences.

**Table 1.** Professional background, practice characteristics, and buprenorphine prescribing by practice setting and prescribing status.

**Practice Setting**	Emergency Department (N = 87)		Other (N = 325)		Totals (N = 412)	sig
**Prescribing Status**	Inactive (N = 25)	Active (N = 62)	Inactive (N = 90)	Active (N = 235)		
**Region^a^**						NS
Chicago/surrounding metropolitan area	83.9%	71.8%	79.6%	77.9%	77.7%	
Greater Illinois	16.1%	28.2%	20.4%	22.1%	22.3%
**Practice Location^a^**						NS
Rural	5.0%	5.1%	4.3%	5.5%	5.2%
Suburban	47.9%	51.4%	36.0%	32.3%	37.0%
Urban	47.1%	43.6%	59.7%	62.2%	57.8%
**DATA X-waiver patient limits^a^**						***
30	59.9%	47.4%	58.6%	38.6%	45.6%
100	12.8%	18.0%	6.4%	30.4%	22.2%
275	0.0%	2.6%	1.2%	18.3%	11.0%
Don't know/unsure	27.3%	32.1%	33.8%	12.7%	21.3%
**Prescribed buprenorphine for OUD**						***
Past-year^**a**^	0.0	100.0%	0.0	100.0%	72.2%	NE
Past-month^**b**^	-	43.3%	-	79.1%	71.3%	***
**MOUD patients as practice percentage in past-year^b^**						NS
0%–25%		94.0%	82.2%	84.8%	
26%–50%		2.7%	10.3%	8.6%	
51%–75%		0.0%	2.5%	1.9%	
76%–100%		3.3%	5.1%	4.7%	
**MOUD patients by age^b^**				
Adolescents (13–17)		10.0%		3.4%	4.8%	NS
Young adults (18–25)		73.7%		72.4%	72.7%	NS
Adults (25–64)		98.5%		98.7%	98.7%	NS
Older adults (65+)		46.6%		56.8%	54.6%	NS
**MOUD patients by race/ethnicity^b^**					
White		98.3%		96.5%	96.9%	NS
Latino/Latinx/Hispanic		92.6%		81.8%	84.3%	NS
Black or African American		92.4%		85.8%	87.3%	NS
American Indian or Alaska Native		45.4%		19.0%	24.8%	***
Asian		49.8%		26.6%	31.7%	***
Native Hawaiian or Pacific Islander		43.7%		16.5%	22.4%	***
**MOUD patient populations^b^**					
Homeless/unstably housed		73.5%		58.7%	61.9%	NS
Criminal justice involved		44.0%		47.6%	46.8%	NS
Maternal/fetal		18.7%		32.1%	29.2%	NS
Undocumented immigrants		39.3%		24.4%	27.7%	NS
Immigrants		41.8%		30.6%	33.0%	NS
LGBTQ+		50.6%		43.7%	45.2%	NS

Note: All figures shown are percentages based on the weighted responses of X-waivered physicians practicing in Illinois in 2021. Significance levels are based on Pearson design-based F tests. OUD = opioid use disorder; MOUD = medication for treating opioid use disorder. ^a^Figures and significance tests based on the full sample of 412 surveyed physicians; ^b^Figures and significance tests based on the subset of 297 surveyed physicians who indicated they had prescribed buprenorphine in the past year; NE = not estimated; NS = non-significant; **p ≤ 0.01; *** p ≤ 0.001.

**Table 2.** Provider barriers to prescribing buprenorphine as MOUD by practice setting and prescribing status.

Practice Setting	Emergency Department (N = 87)		Other (N = 325)			OLS Regression Results					
Prescribing Status	Inactive (N = 25)	Active (N = 62)	Inactive (N = 90)	Active (N = 235)	Totals (N = 412)	ED/Other Setting		Active/Inactive Prescribing		Setting by Prescribing Status	
Provider Barriers	Mean (SE)	Mean (SE)	Mean (SE)	Mean (SE)	Mean (SE)	b [95% CI]	sig	b [95% CI]	sig	b [95% CI]	sig
Providers who offer MOUD services are often stigmatized	2.2 (0.17)	2.0 (0.12)	2.4 (0.10)	2.3 (0.07)	2.3 (0.05)	−0.6 [−0.56, 0.22]	NS	−0.1 [−0.34, 0.16]	NS	−1.1 [−0.59, 0.36]	NS
Patients who do not receive MOUD services do not want to see a doctor who provides MOUD services to others	2.4 (0.13)	2.5 (0.13)	2.5 (0.09)	2.3 (0.06)	2.4 (0.05)	−0.1 [−0.38, 0.25]	NS	−0.2 [−0.44, 0.02]	NS	0.3 [−0.13, 0.73]	NS
There are insufficient community-based MOUD treatment providers	3.6 (0.18)	3.4 (0.13)	3.7 (0.09)	3.6 (0.07)	3.6 (0.05)	−0.1 [−0.54, 0.26]	NS	−0.06 [−0.28, 0.16]	NS	−0.1 [−0.65, 0.35]	NS
My practice does not support MOUD providers	2.7 (0.22)	2.0 (0.13)	2.9 (0.14)	1.9 (0.06)	2.2 (0.06)	−0.2 [−0.73, 0.29]	NS	−0.94 [−1.24, −0.63]	***	0.3 [−0.33, 0.84]	NS
Insufficient behavioral health treatment referral sources (e.g., addiction counselors) to support patients receiving MOUD services	4.0 (1.8)	3.9 (0.12)	4.2 (0.09)	3.9 (0.07)	3.9 (0.05)	−0.2 [−0.58, 0.21]	NS	−0.3 [−0.54, −0.08]	**	0.2 [−0.27, 0.71]	NS
My current caseload is too large to see new patients that receive MOUD services	2.4 (0.16)	2.2 (0.12)	3.3 (0.12)	2.6 (0.08)	2.7 (0.06)	−0.9 [−1.26, −0.45]	NS	−0.7 [−1.00, −0.41]	NS	0.5 [−0.04, 0.94]	NS
Lack of knowledge about insurance coverage for MOUD services	3.5 (0.12)	3.4 (0.12)	3.6 (0.09)	3.4 (0.07)	3.4 (0.05)	−0.1 [−0.40, 0.19]	NS	−0.2 [−0.41, 0.11]	NS	0.1 [−0.30, 0.50]	NS
Insurance does not provide sufficient reimbursement to cover the cost of providing MOUD services	3.1 (0.15)	2.9 (0.11)	3.1 (0.08)	3.2 (0.07)	3.2 (0.05)	−0.5 [−0.39, 0.28]	NS	0.8 [−0.13, 0.28]	NS	−0.2 [−0.64, 0.19]	NS
Lack of medical school training or continuing education opportunities about OUD and MOUD topics	2.9 (0.06)	2.9 (0.11)	3.1 (0.07)	3.0 (0.06)	3.0 (0.04)	−0.2 [−0.33, 0.14]	NS	−0.6 [−0.23, 0.12]	NS	0.1 [−0.23, 0.37]	NS
There is a delay in receiving insurance reimbursement	3.7 (0.16)	3.6 (0.13)	3.8 (0.10)	3.6 (0.7)	3.6 (0.05)	−0.1 [0.47, 0.27]	NS	−0.2 [−0.5, 0.02]	NS	0.12 [−0.35, 0.59]	NS

Note: Figures shown are means and standard errors or OLS regression coefficients and 95% confidence intervals. For the OLS regressions, each provider barrier was the dependent variable with predictors for practice, prescribing status, and their interaction. All models are based on the weighted responses of 412 Illinois physicians with X-waivers as of 2021. Significance levels for the regression models are based on t-tests. OUD = opioid use disorder; MOUD = medication for treating an opioid use disorder. NS = non-significant; **p ≤ 0.01; *** p ≤ 0.001.

### 3.3. Practice expansion barriers

Among physicians who indicated that they had not prescribed buprenorphine in the past year (Table 3), the most frequently endorsed barriers were: Difficulties building systems to support this work (45.1%); challenges around knowing where or how to refer for behavioral support services (32.2%); insufficient time to take on a new area of practice (30.8%); and limited demand for services (28.4%). Physicians in ED-based settings were much more likely than physicians in other practice settings to endorse not knowing where to refer MOUD patients for behavioral support services (58.2% vs. 25.2%, F_(1, 644)_ = 8.84, p = 0.003) and much less likely to endorse having insufficient time for a new area of practice (6.0% vs. 37.4%, F_(1, 644)_ = 14.1, p < 0.001). No other comparisons were statistically significant.

### 3.4. Training needs

Only one of the comparisons of practice setting and prescribing status was statistically significant with respect to specific training needs (Table 4); active prescribers were less likely to endorse needing training on skill building and practice in prescribing (aOR = 0.41, CI = 0.22–0.77, p < 0.01). The non-significance of the remaining comparisons indicates broad agreement about the trainings needed among survey participants. In descending order, the five most frequently endorsed training needs were: Medication dosing (61.9%); prescribing to patients with comorbidities (60.5%); detox regimen before administering buprenorphine (59.9%); skill building and practice in prescribing (56.7%); and incorporating external resources such as therapy and psychiatry into training plans (52.5%).

**Table 3.** Barriers to buprenorphine prescribing among inactive providers by practice setting.

Practice Setting	Emergency Department (N = 25)	Other (N = 90)	Totals (N = 115)	sig
Barriers to Prescribing				
Insufficient education/background for providing care for people with OUD	26.9%	21.1%	22.3%	NS
Insufficient support from my employer	26.6%	17.2%	19.2%	NS
Challenges around insurance and prior authorizations	12.3%	9.0%	9.7%	NS
Challenges around knowing where or how to refer for behavioral support	58.2%	25.2%	32.2%	**
Difficulties building systems to support this work	49.9%	43.8%	45.1%	NS
Insufficient time to take on a new area of practice	6.0%	37.4%	30.8%	***
Limited demand for services	47.2%	23.3%	28.4%	NS
Poor experiences when working with patients with addiction in the past	12.2%	8.1%	9.0%	NS

Note: All figures shown are percentages based on the weighted responses of 115 Illinois-based physicians with X-waivers who indicated they had not prescribed buprenorphine in the past year. Significance levels are based on design-based F tests. OUD = opioid use disorder; NS = non-significant. **p ≤ 0.01; *** p ≤ 0.001.

**Table 4.** Trainings needed to support prescribing bupreneorphine as MOUD by practice setting and prescribing status.

	Emergency Department (N = 87)		Other Practice Setting (N = 325)			Logistic Regression Results					
Trainings Needed	Inactive (N = 25)	Active (N = 62)	Inactive (N = 90)	Active (N = 235)	Totals (N = 412)	ED/Other Setting		Active/Inactive Prescribing		Practice Setting by Prescribing Interaction	
						OR [95% CI]	sig	OR [95% CI]	sig	OR [95% CI]	sig
Skill building and practice in prescribing	41.9%	44.7%	74.1%	54.1%	56.7%	0.25 [0.09, 0.72]	NS	0.41 [0.22, 0.77]	**	2.71 [0.76, 9.74]	NS
Medication dosing	79.4%	72.3%	68.9%	54.5%	61.9%	1.73 [0.30, 5.33]	NS	0.54 [0.30, 0.99]	NS	1.25 [0.29, 5.33]	NS
Prescribing to patients with comorbidities	53.4%	70.6%	63.8%	60.5%	60.5%	0.65 [0.23, 1.81]	NS	0.87 [0.48, 1.58]	NS	2.40 [0.66, 8.70]	NS
Detox regimen before administering buprenorphine	64.5%	55.0%	65.2%	59.9%	59.9%	0.97 [0.33, 2.80]	NS	0.80 [0.44, 1.44]	NS	0.85 [0.23, 3.06]	NS
Method of medication administration (e.g., injectable, oral, transplant)	5.0%	15.6%	43.0%	50.4%	41.8%	0.07 [0.01, 0.55]	NS	1.35 [0.76, 2.39]	NS	2.62 [0.27, 25.84]	NS
Psychosocial support for patients	19.2%	42.2%	45.7%	49.6%	49.6%	0.28 [0.08, 0.96]	NS	1.17 [0.66, 2.08]	NS	2.61 [0.63, 10.87]	NS
Incorporating external resources (e.g., therapy, psychiatry) into treatment plans)	39.3%	31.7%	41.2%	52.5%	52.5%	0.93 [0.33, 2.59]	NS	1.58 [0.89, 2.81]	NS	0.45 [0.12, 1.64]	NS
Specialized training for different settings and/or different provider types	30.2%	27.0%	38.8%	32.9%	32.9%	0.68 [0.23, 2.03]	NS	0.77 [0.43, 1.40]	NS	1.10 [0.28, 4.37]	NS
Concerns around Drug Enforcement Administration (DEA) or other legal concerns	18.1%	21.8%	31.3%	29.9%	29.9%	0.48 [0.14, 1.65]	NS	0.93 [0.50, 1.73]	NS	1.34 [0.30, 6.08]	NS

Note: All figures shown are percentages or odds ratios with associated 95% confidence intervals. Odds ratios were estimated using binary logistic regression with each barrier as the dependent variable and predictors for practice, prescribing status, and their interaction. All models are based on the weighted responses of 412 Illinois physicians with X-waivers as of 2021. Significance levels are based on design-based F tests. OUD = opioid use disorder; MOUD = medication for treating opioid use disorder. NS = non-significant; **p ≤ 0.01; *** p ≤ 0.001.

**Table 5.** Additional supports needed to increase buprenorphine prescribing by practice setting and prescribing status.

	Emergency Department (N = 87)		Other Practice Setting (N = 325)			Logistic Regression Results					
	Inactive (N = 25)	Active (N = 62)	Inactive (N = 90)	Active (N = 235)	Totals (N = 412)	ED/Other Setting		Active/Inactive Prescribing		Practice Setting by Prescribing Interaction	
						OR [95% CI]	sig	OR [95% CI]	sig	OR [95% CI]	sig
Administrative support	37.7%	42.7%	45.7%	39.8%	41.4%	0.72 [0.29, 1.79]	NS	0.78 [0.48, 1.29]	NS	1.57 [0.53, 4.63]	NS
Case management support	58.3%	64.2%	52.8%	54.7%	56.0%	1.25 [0.50, 3.11]	NS	1.08 [0.66, 1.77]	NS	1.18 [0.40, 3.49]	NS
Better understanding of support and resources available	67.0%	41.9%	36.0%	34.2%	37.7%	3.62 [1.39, 9.38]	**	0.92 [0.55, 1.54]	NS	0.38 [0.13, 1.17]	NS
Assistance in navigating referrals	58.7%	46.6%	30.0%	28.7%	33.5%	3.37 [1.32, 8.56]	NS	0.95 [0.55, 1.64]	NS	0.64 [0.21, 1.93]	NS
Support network of other prescribers and mentors	31.1%	23.0%	34.2%	29.5%	29.6%	0.87 [0.33, 2.27]	NS	0.80 [0.48, 1.35]	NS	0.82 [0.26, 2.62]	NS
Increased MOUD prescribing capacity for other providers in my practice	12.5%	12.6%	15.9%	23.2%	19.3%	0.76 [0.19, 2.91]	NS	1.60 [0.84, 3.07]	NS	0.63 [0.13, 3.08]	NS
No other supports are necessary	12.7%	15.6%	12.7%	18.5%	16.4%	0.99 [0.25, 3.88]	NS	1.55 [0.77, 3.12]	NS	0.82 [0.17, 3.89]	NS

Note: All figures shown are percentages or odds ratios and associated 95% confidence intervals. Odds ratios were estimated using binary logistic regression with each barrier as the dependent variable and predictors for practice location, prescribing status, and their interaction. All figures are based on the weighted responses of 412 Illinois physicians with X-waivers as of 2021. MOUD = medication for treating opioid use disorder; NS = non-significant; **p ≤ 0.01; *** p ≤ 0.001.

### 3.5. Needed support for practice expansion

As with trainings needed, there was broad agreement among the surveyed physicians on the kinds of supports needed to expand buprenorphine prescribing (Table 5). There was only one statistically significant difference, with ED-based physicians more likely to indicate they wanted a better understanding of the support and resources available (aOR = 3.62, CI = 1.39–9.38, p < 0.01) compared with physicians in other practice settings. Of the 7 items considered, only case management support (56.0%) was endorsed by more than half of the participants, followed by administrative support (41.4%), having a better understanding of support and resources available (37.7%), and assistance in navigating referrals (33.5%).

## 4. Discussion

Contrary to expectations, we did not find many statistically significant differences in perceived barriers to expanding buprenorphine prescribing to treat OUD by practice setting. The single significant difference was that ED-based physicians saw current caseload size as a less important barrier. Otherwise, ED-based physicians and those practicing in other settings prioritized the same set of barriers. These included having adequate treatment resources in the community for referring patients, insurance coverage, needing more training in medical school, and needing continuing education opportunities for treating patients with an OUD. We believe for ED-based physicians, having or knowing about available referral resources could mean having a medical practice to which they could refer those inducted on buprenorphine to continue the course of MOUD whereas for physicians practicing in other settings, having a referral network is more likely to mean having behavioral healthcare and case management services available as wrap-around services for the ongoing MOUD treatment they provide.

There were also some significant differences by prescribing status regardless of practice setting. Compared with active prescribers, inactive prescribers rated the following as more important barriers to prescribing: their practice did not support MOUD providers, having insufficient behavioral health treatment referral resources, and having too large a current caseload to expand MOUD treatment.

Examining barriers to prescribing among the subset of participants who indicated that they had not prescribed buprenorphine in the past month, ED-based physicians were more likely to endorse challenges around knowing where or how to refer patients for behavioral support whereas physicians practicing in other settings were much more likely to endorse they had insufficient time to take on a new area of practice. Otherwise, the most frequently cited barrier to prescribing regardless of practice setting was the difficulties building systems to support this work, possibly also a reflection of the need for having an adequate referral network and an available system for managing referrals. We note that the lack of community resources for supporting MOUD care was a theme that surfaced repeatedly in the qualitative interviews conducted for the SUPPORT study [8].

There were no significant differences by either practice setting or prescribing status with respect to the trainings or supports needed. In terms of specific trainings, those most frequently identified were prescribing to patients with comorbidities, understanding the detox regimen before administering buprenorphine, medication dosing, and skill building and practice prescribing. Consistent with concerns over having treatment and other resources available in the community, the most frequently endorsed support needed was for case management services. The one significant difference between ED-based and physicians in other practice settings was that ED-based physicians were more likely to indicate that they needed a better understanding of the support and resources available.

We obtained data from practitioners who had obtained an X-waiver to prescribe buprenorphine, which was required at the time of the survey. Therefore, we do not know how well the results apply to those who can now prescribe buprenorphine as MOUD without having to obtain an X-waiver or without patient limits. We also could not determine if the identified barriers and supports would apply to physicians who were not waivered at the time and not prescribing buprenorphine as MOUD. Therefore, we do not know why some providers did not get an X-waiver and were not interested in prescribing buprenorphine for treating an OUD. As the survey was administered to Illinois physicians and state laws vary with respect to buprenorphine-prescribing practice restrictions, the results also might not apply to physicians practicing in other states. Finally, our response rate among all healthcare providers we attempted to contact was low, about 25%. Thus, there is potential bias owing to differences between those who did and did not participate in the study.

## 5. Conclusions

Regardless of practice setting, the most frequently perceived barriers to and the supports needed for increasing buprenorphine prescribing have to do with having an adequate community-based behavioral health treatment system in place to provide the additional care needed by patients with an OUD receiving medication. Patients with an OUD often have a complex set of psychosocial needs that can't be addressed by medication alone [33]. Recognizing this, physicians and other healthcare providers might be reluctant to prescribe buprenorphine for treating OUD if they do not have available referral sources or do not know how to provide referrals to an established, robust network of community-based behavioral healthcare treatment providers for care coordination. By extension, having a case management system available to manage and monitor treatment referrals post-MOUD induction is also important. Physicians working in an ED or elsewhere do not have the time or expertise to make and monitor treatment referrals.

Based on our findings, we recommend that efforts to expand buprenorphine for OUD focus as much on providing additional supports to make and manage referrals and expand the availability of community-based behavioral healthcare services as on recruiting additional providers. Building supportive networks of care could have as much if not more impact on expanding the availability of MOUD as increasing the number of practitioners trained and willing to prescribe buprenorphine.

## Use of AI tools declaration

The authors declare they have not used Artificial Intelligence (AI) tools in the creation of this article.


