# Supplementary material for: Barriers and facilitators to prescribing buprenorphine for treating opioid use disorder among emergency department and other practice setting physicians
Source: AIMS Public Health. 2025 Jan 9;12(1):56–76. doi: 10.3934/publichealth.2025005 (PMC11999815; doi:10.3934/publichealth.2025005)
Supplement: Supplementary file 1 [file publichealth-12-01-005-s001.pdf]

AIMS Public Health

AIMS Public Health, 12(1): 56–76.
DOI: 10.3934/publichealth.2025005
Received: 16 May 2024
Revised: 03 July 2024
Accepted: 23 August 2024
Published: 09 January 2025

https://www.aimspress.com/journal/aimsph

Research article

Barriers and facilitators to prescribing buprenorphine for treating opioid use disorder among emergency department and other practice setting physicians

James A. Swartz[1,*], Dana Franceschini[1], Nora M. Marino[2], Adrienne H. Call[2], Lisa Rosenberger[2] and Sarah Whitehouse[2]

[1] Jane Addams College of Social Work, University of Illinois Chicago, 1040 W. Harrison St., MC (309), Chicago IL 60607, USA
[2] National Opinion Research Center at the University of Chicago, 55 East Monroe St, 30th Floor, Chicago IL 60603, USA

* **Correspondence:** Email: jaswartz@uic.edu; Tel: +3129968560.

Supplementary

Buprenorphine Provider Survey 2021 – IL SUPPORT

In partnership with the University of Illinois Chicago and the Illinois Department of Healthcare and Family Services, NORC at the University of Chicago is conducting a survey of Illinois providers who have experience diagnosing and/or treating patients diagnosed with opioid use disorder (OUD). You are listed in the Illinois buprenorphine prescriber registry.

The survey seeks to understand your experiences in working with patients with OUD and/or providing medication-assisted recovery (MAR), also referred to as medication-assisted treatment (MAT) or medication for opioid use disorder (MOUD). We hope this information will help the state of Illinois work to improve access to treatment and treatment services for individuals with OUD. The survey will last about 10 minutes. **To thank you for your participation, you will receive a $50 Visa electronic gift card after completing the survey.**

It’s your choice to complete this voluntary survey. All collected data will be analyzed in aggregate and will not identify individual providers or responses. Your knowledge and experience is important to understanding how to improve services for Illinois Medicaid MCO members with OUD; we hope you choose to assist us in this research by clicking the “Begin Survey” button below.

[start: same page]

Section I: Professional Background and Practice Characteristics

In this section we will ask you questions about your professional background and the characteristics of your primary practice. [transition1]

1. Please think about the practice where you do the majority of your buprenorphine prescribing. What is the city or town and zip code for this practice?

1. City/Town: *[open text]* *[q1a]*
2. Zip/Postal Code *[open text that only allows for 5-digit numbers]* *[q1b]*

[end: same page]

2. Please select your credential: *[select one]* *[q2]*

1. MD/DO
2. PA
3. NP
4. Other

3a. In what specialty do you primarily practice? *[select one]* *[q3a]*

1. Addiction Medicine
2. Addiction Psychiatry
3. Adult Geriatrics
4. Emergency Medicine
5. Family Medicine
6. General Practitioner
7. Internal Medicine
8. Hospitalist
9. Neurology
10. OB/GYN
11. Pain Management
12. Palliative Medicine
13. Pediatrics
14. Psychiatry
15. Urgent Care

16. Other (Please specify specialty in the textbox below)

If q3a = 16, show q3b, else q4

3b. Please indicate your specialty. *[open textbox] [q3b]*

4. Do you serve patients insured through Medicaid? *[select one] [q4]*

1. Yes
2. No

If q4 = 1, show q5, else q6

5. If yes, please indicate which Medicaid plans you accept. (Select all that apply) *[select all] [q5]*

1. Aetna Better Health of Illinois
2. Blue Cross/Blue Shield
3. CountyCare Health Plan
4. Meridian Health
5. Molina Healthcare
6. YouthCare Managed Care Organizations (MCO) plans outside of Illinois
7. Medicaid Fee-for-Service

77. Don't know

[start: same page]

Section II: Prescribing Practices

In this section we will ask about your prescribing practices of buprenorphine for OUD treatment. The following questions **do not** pertain to any prescribing of buprenorphine as an analgesic for treating pain. [transition2]

6a. In the past year, have you prescribed buprenorphine to any of your patients to treat OUD? *[q6a]*

1. Yes
2. No

[end: same page]

If q6a = 1, show 6b

6b. In the past month, have you prescribed buprenorphine to any patients? *[q6b]*

1. Yes
2. No

If q6a = 1, go to q9

If q6a = 2, go to q7, q8 and then go to q17a

7. Do you have any plans to start prescribing buprenorphine to treat OUD? *[select one] [q7]*

1. Yes
2. No

8a. Which of the following reasons prevent you now, or prevented you in the past from prescribing buprenorphine for treating OUD? (Select all that apply) *[select all] [q8a]*

1. Insufficient education/background in providing care for people with OUD
2. Insufficient support from my employer
3. Challenges around insurance and prior authorizations
4. Challenges around knowing where or how to refer for behavioral support
5. Difficulties building systems to support this work
6. Insufficient time to take on a new area of practice
7. Limited demand for services
8. Poor experiences when working with patients with addiction in past
9. Concerns around Drug Enforcement Administration (DEA) or other legal concerns
10. Other (please specify)

If q8a = 10, show q8b

8b. Please describe any other reasons. *[open textbox] [q8b]*

9. Are you accepting new patients with OUD? *[select one] [q9]*

1. Yes
2. No

If q9 = 2, show 10

10a. What are the reasons you are not currently accepting new patients with OUD? (Select all that apply) *[select all] [q10a]*

1. No capacity based on current limit or treatment maximum.
2. Do not want medication-assisted recovery (MAR) to encompass more than a certain percentage of my total clinical time.
3. Considering leaving my practice/retiring in near future.
4. Insufficient support from my employer regarding expanding OUD treatment.
5. Challenges around insurance and prior authorizations
6. Challenges around knowing where to refer for behavioral support
7. Concerns around DEA or other legal issues
8. Difficulties building systems to support this work

9. Working with patients with addiction can be very time consuming in terms of the amount of support and case management needed
10. Other (please specify)

If q10a = 10, show q10b, else q11

10b. Please describe any other reasons. *[textbox] [q10b]*

11. In the past year, approximately <u>what percentage of your time</u> practicing (across all applicable settings) is dedicated to treating patients with OUD with buprenorphine? *[select one] [q11]*

1. 0–25% of my time
2. 26–50% of my time
3. 51–75% of my time
4. 76–100% of my time

77. Don't know

12. In the past year, to approximately <u>what percentage of your patients</u> are you currently prescribing buprenorphine for OUD treatment? *[select one] [q12]*

1. 0–25% of my patients
2. 26–50% of my patients
3. 51–75% of my patients
4. 76–100% of my patients

77. Don't know

13. Please indicate the age of the populations for which you prescribe buprenorphine for OUD treatment. (Select all that apply) *[select all] [q13]*

1. Children, 2–12 years
2. Adolescents, 13–17 years
3. Young Adults, 18–24 years
4. Adults, 25 years to 64 years
5. Older Adults, 65 and older

14. Please indicate the race of the populations for which you prescribe buprenorphine for OUD treatment. (Select all that apply) *[select all] [q14]*

1. White
2. Black or African American
3. American Indian or Alaska Native
4. Asian
5. Native Hawaiian or Pacific Islander
6. Other

15. Do you prescribe buprenorphine for OUD treatment to populations who are of Hispanic Latino/x, or Spanish origin? *[select one] [q15]*

1. Yes
2. No
77. Don’t know

16a. Please indicate any patient population for which you prescribe buprenorphine for OUD treatment. (Select all that apply) *[select all] [q16a]*

1. Urban
2. Rural
3. Suburban
4. Homeless/unstable housing
5. Criminal justice involved
6. Maternal/fetal
7. Undocumented immigrants
8. Immigrants
9. LGBTQIA
10. Other, please specify

If q16a = 10, show q16b, else q17a

16b. Please specify the patient population. *[open textbox] [q16b]*

17a. In what setting(s) do you prescribe buprenorphine for OUD treatment? If you work in multiple settings, please select all that apply. *[select all] [q17a]*

1. Independent private practice (outpatient)
2. Group private practice (outpatient)
3. Federally qualified health center (outpatient)
4. Residential treatment facility
5. Opioid treatment program (OTP)
6. Medical stabilization unit (Detox Unit)
7. Community hospital
8. Academic hospital
9. Private hospital
10. Locum tenens/temporary position at different locations
11. Other

If q17a = 7, 8, or 9, show q17b, else q18a

17b. You indicated that you work in a hospital setting. Are you an ER doctor? *[q17b]*

1. Yes

2. No

18a. Did you participate in an incentive program to get your X-waiver for prescribing buprenorphine for OUD? *[select one] [q18a]*

1. Yes
2. No

If q18a = 1, show q18b, else go q19a

18b. What was the name of the incentive program in which you participated? *[text box] [q18b]*

19a. Have you given consent to be listed in the Substance Abuse and Mental Health Services Administration (SAMHSA) treatment locator as a buprenorphine provider? *[select one] [q19a]*

1. Yes
2. No

If q19a = 2, show q19b, else q20

19b. What is/are the reason(s) you have not given consent to be listed publicly in the SAMHSA locator as a buprenorphine provider? *[textbox] [q19b]*

20. According to your X-waiver, how many patients are you allowed to treat with buprenorphine? *[select one] [q20]*

1. 30 patients maximum
2. 100 patients maximum
3. 275 patients maximum

77. Not sure

21a. Do you plan on submitting a request to increase the number of patients for whom you can prescribe buprenorphine for OUD treatment? *[select one] [q21a]*

1. Yes
2. No

If q21a = 2, show q21b, else go q22

21b. Why not? *[textbox] [q21b]*

[start: same page]

Section III: Barriers

Show text: In this section we will ask about any barriers you have encountered in prescribing buprenorphine for OUDs or delivering medication-assisted recovery (MAR) (also known as medication-assisted treatment (MAT)). [transition3]

22. Do you feel that you have a good understanding of medication-assisted recovery (MAR) best practices for treating OUD? *[select one] [q22]*

1. Yes
2. No

[end: same page]

23. Do you feel that additional training on MAR for OUD would be helpful? *[select one] [q23]*

1. Yes
2. No

If q23 = 1, show q24a, else go q25a

24a. What specific MAR training topics would be helpful to you or your practice? (Select all that apply) *[select all] [q24a]*

1. Skill building and practice in prescribing
2. Medication dosing
3. Prescribing to patients with comorbidities
4. Detox regimen before administering buprenorphine
5. Method of medication administration (i.e., injectable, oral, transplant)
6. Psychosocial support for patients
7. Incorporating external resources (therapy, psychiatry) into treatment plans
8. Specialized training for different settings and/or different provider types
9. Review of X-waiver requirements and restrictions
10. Other, please specify

If q24a = 10, show q24b, else q25a

24b. Please include other topics that would be helpful to you. *[open textbox] [q24b]*

25a. What, if any, additional support would be helpful to increase your MAR prescribing capacity? (Select all that apply) *[select all] [q25a]*

1. Administrative support
2. Case management support
3. Better understanding of support and resources available
4. Assistance in navigating referrals
5. Support network of other prescribers and mentors
6. Increased MAR prescribing capacity for other providers in my practice
7. Access to updated X-waiver policies and information
8. No other supports are necessary [exclusive response]
9. Other, please specify

If q25a = 9, show q25b, else q26

25b. Please include other supports that would be helpful. *[open textbox] [q25b]*

26. The following is a list of barriers that patients may face when seeking MAR services for OUDs. Please indicate your level of agreement that the following are barriers to seeking MAR services for OUD. *[grid, select one, randomize grid items] [q26]*

Question	*Answer choices, select one*				
Insufficient number of MAR providers in the community *[q26a]*	1. Strongly Disagree	2. Disagree	3. Neutral	4. Agree	5. Strongly Agree
Insufficient number of mental health providers in the community (i.e. social workers or addiction counselors) *[q26b]*	1. Strongly Disagree	2. Disagree	3. Neutral	4. Agree	5. Strongly Agree
Inadequate insurance coverage or lengthy authorization for MAR services *[q26c]*	1. Strongly Disagree	2. Disagree	3. Neutral	4. Agree	5. Strongly Agree
Serious mental illness (SMI) *[q26d]*	1. Strongly Disagree	2. Disagree	3. Neutral	4. Agree	5. Strongly Agree
Unstable access to housing *[q26e]*	1. Strongly Disagree	2. Disagree	3. Neutral	4. Agree	5. Strongly Agree
Unreliable transportation to receive MAR services *[q26f]*	1. Strongly Disagree	2. Disagree	3. Neutral	4. Agree	5. Strongly Agree

Distance from MAR treatment facilities *[q26g]*	1. Strongly Disagree	2. Disagree	3. Neutral	4. Agree	5. Strongly Agree
PAGE SPLIT					
Distance from a pharmacy that fills MAR prescriptions (i.e., a 340b pharmacy that is contracted with the Centers for Medicare & Medicaid) *[q26h]*	1. Strongly Disagree	2. Disagree	3. Neutral	4. Agree	5. Strongly Agree
Pharmacists' refusal to fill MAR prescriptions *[q26i]*	1. Strongly Disagree	2. Disagree	3. Neutral	4. Agree	5. Strongly Agree
Fear of stigmatization for seeking or receiving MAR *[q26j]*	1. Strongly Disagree	2. Disagree	3. Neutral	4. Agree	5. Strongly Agree
Doctors' refuse to offer MAR services *[q26k]*	1. Strongly Disagree	2. Disagree	3. Neutral	4. Agree	5. Strongly Agree
Lack of community support for individuals who receive MAR (i.e., family or friends; religious or community groups) *[q26l]*	1. Strongly Disagree	2. Disagree	3. Neutral	4. Agree	5. Strongly Agree
Other barriers *[q26m]*	1. Strongly Disagree	2. Disagree	3. Neutral	4. Agree	5. Strongly Agree

If q26l = 4 or 5, show 26m, else follow logic from q4 to show q27 and/or q29

26n. Please describe other barriers: *[textbox] [q26n]*

If q4 = 1, show q27, else 29

27. Have you or the practice/setting in which you work experienced any difficulty in receiving reimbursement for a Medicaid or Medicaid MCO patient visit where you prescribed or administered buprenorphine? *[select one] [q27]*

1. Yes
2. No
77. Don't know

If q27 = 1, show q28, else q29

28. Please explain the reason(s) it was difficult to receive reimbursement. *[textbox] [q28]*

29. The following is a list of barriers that providers may face when administering MAR services. Please indicate your level of agreement that the following are barriers to administering MAR services. *[grid, select one, randomize grid items] [q29]*

Question	*Answer choices, select one*				
Providers who offer MAR services are stigmatized *[q29a]*	1. Strongly Disagree	2. Disagree	3. Neutral	4. Agree	5. Strongly Agree
Patients who do not receive MAR do not want to see a doctor that provides MAR services *[q29b]*	1. Strongly Disagree	2. Disagree	3. Neutral	4. Agree	5. Strongly Agree
Insufficient MAR providers in the community *[q29c]*	1. Strongly Disagree	2. Disagree	3. Neutral	4. Agree	5. Strongly Agree
My practice does not support MAR providers *[q29d]*	1. Strongly Disagree	2. Disagree	3. Neutral	4. Agree	5. Strongly Agree
Insufficient behavioral health treatment referral sources to support my patients that receive MAR services (i.e., addiction counselors) *[q29e]*	1. Strongly Disagree	2. Disagree	3. Neutral	4. Agree	5. Strongly Agree
My current caseload is too large to see new patients that receive MAR services *[q29f]*	1. Strongly Disagree	2. Disagree	3. Neutral	4. Agree	5. Strongly Agree
PAGE SPLIT					
Lack of knowledge about insurance coverage for MAR services *[q29g]*	1. Strongly Disagree	2. Disagree	3. Neutral	4. Agree	5. Strongly Agree
Insurance does not provide sufficient reimbursement to cover the cost of providing MAR services*[q29h]*	1. Strongly Disagree	2. Disagree	3. Neutral	4. Agree	5. Strongly Agree
There is a delay in receiving insurance reimbursement for MAR services *[q29i]*	1. Strongly Disagree	2. Disagree	3. Neutral	4. Agree	5. Strongly Agree
Lack of medical school training or continuing education opportunities about OUD and MAR topics *[q29j]*	1. Strongly Disagree	2. Disagree	3. Neutral	4. Agree	5. Strongly Agree
Other barriers *[q29k]*	1. Strongly Disagree	2. Disagree	3. Neutral	4. Agree	5. Strongly Agree

If q29j = 4 or 5, show q29l, else q30a

29l. Please describe other barriers: *[textbox] [q29l]*

30a. From whom do you receive support for MAR services? (Select all that apply) *[select all] [q30a]*

1. Medical support staff (medical assistants, etc.)
2. Administration
3. Nurses (NP/RN)
4. PAs
5. Collaborating physician (MD/DO/NP/RN/PA)
6. Other, please describe
7. I don't receive support for MAR services

If q30a = 6, show q30b, else q31

30b. Please describe: *[textbox] [q30b]*

31. Have you experienced problems in referring your patients who receive MAR to other addiction treatment services? *[select one] [q31]*

1. Yes
2. No

32a. Does being a MAR provider have a negative impact on your productivity requirements (e.g., increased no-show rates, requires extra time, etc.)? *[select one] [q32a]*

1. Yes
2. No

If q32a = 1, show q32b

32b. Please explain. *[textbox] [q32b]*

[start: same page]

Section IV: What would make it better?

We are committed to exploring new ways to encourage buprenorphine prescribing to treat OUD. Additionally, we are interested in determining what would help X-Waivered prescribers increase their prescribing capacity. Feedback from the medical community about how to expand MAR and buprenorphine treatment for OUDs across Illinois will help us better understand where improvements can be made. [transition4]

If q20 = 1 or 2 show q33a, else q34

33a. Please indicate below what would help increase your capacity to treat additional patients with MAR. (Select all that apply) *[select all] [q33a]*

1. Technical assistance for expanding the systems to carry out MAR and OUD treatment in my practice
2. In-person educational events to provide continued learning on MAR and treating patients with OUD
3. Online educational (e.g., webinars) events to provide continued learning on MAR and treating patients with OUD
4. State-level hotline resources for providers to ask questions about MAR services and to learn about community resources
5. Access to an addiction specialist who can answer clinical questions
6. Assistance with identifying local addiction treatment programs where my patients can receive counseling and case management services
7. Financial support/grants to support a nurse care manager or similar position in my practice
8. Guidance on working with my practice to gain support to implement MAR services in our clinical setting
9. I do not want to increase the number of patients with OUD/buprenorphine I see beyond my current waivered limit
10. My practice does not have the demand to support a waiver increase
11. Other (please specify)

[end: same page]

If q33a = 11, show q33b, else q34

33b. Please describe: *[textbox] [q33b]*

34. What short-term (6-24 months) steps could the state take to help increase MAR provider capacity, thereby increasing the number of individuals receiving MAR? *[textbox] [q34]*

35. What longer-term (2-10 years) steps could the state take to help increase MAR provider capacity, thereby increasing the number of individuals receiving MAR? *[textbox] [q35]*

[start: same page]

Section V: COVID-19

In this section, we will ask about how the COVID-19 pandemic impacted your ability to provide MAR services and prescribe buprenorphine for OUD. [transition5]

36. During the COVID-19 pandemic did you continue to prescribe buprenorphine? *[select one] [q36]*

1. Yes
2. No

[end: same page]

37. How did the COVID-19 pandemic impact your ability to provide MAR services? *[Open ended]* **[q37]**

38a. During the COVID-19 pandemic, in what setting(s) have you provided MAR services to your patients? Select all that apply. *[select all] [q38a]*

1. Telemedicine visits
2. Urgent care clinic
3. Emergency room
4. Hospital (other than emergency room)
5. In-person office visits
6. Addiction or MAR treatment centers
7. Other location, please describe

If q38a = 7, show q38b, else q39

38b. Other setting: *[textbox]* *[q38b]*

39a. Do you feel comfortable using telemedicine visits to treat patients receiving MAR? *[select one]* *[q39a]*

1. Yes
2. No

If 39a = 2, show q39b

39b. Please explain what challenges telemedicine presents for you. *[textbox]* *[q39b]*

40a. How did the COVID-19 pandemic impact patients' ability to seek MAR services? *[q40a]* *[open ended]*

41. You indicated that the following are barriers that patients face when seeking MAR services for OUD. Please select which barriers, if any, to seeking MAR services for OUD increased because of the COVID-19 pandemic. Select all that apply. *[q41]* *[select all]* [populate items from q26 where respondent selected 4 or 5. If all items were 1, 2, or 3, respondent will skip questions]

1. Insufficient number of MAR providers in the community
2. Insufficient number of mental health providers in the community (i.e. social workers or addiction counselors)
3. Inadequate insurance coverage or lengthy authorization for MAR services
4. Serious mental illness (SMI)
5. Unstable access to housing
6. Unreliable transportation to receive MAR services
7. Distance from MAR treatment facilities

8. Distance from a pharmacy that fills MAR prescriptions (i.e., a 340b pharmacy that is contracted with the Centers for Medicare & Medicaid)
9. Pharmacists' refusal to fill MAR prescriptions
10. Fear of stigmatization for seeking or receiving MAR
11. Doctors' refuse to offer MAR services
12. Lack of community support for individuals who receive MAR (i.e., family or friends; religious or community groups)
13. [text from 26m]
14. None of the above barriers increased because of the COVID-19 pandemic

42. Please include any other information you would like to share with us in the text box below.

[textbox] [q42]

[incentive]

Thank you! You have completed the survey. To thank you for taking the survey, we will email you a $50 Visa electronic gift card. **Please provide an email address below so that we can send you the gift card.** NORC will only use this email address to send you the gift card. We will not share this email with anyone outside of the research team.

Please enter your email: [ins_email1] ______________________________

Please confirm your email: [ins_email2]______________________________

❑ I do not want to provide my email address. I understand that I will view the electronic gift card code on the next page and will need to write the code down. [no_email]

PROMPT IF EMAILS ENTERED ARE DIFFERENT: You provided two different emails, please check that you typed your email correctly.

When you have finished, please select the "Submit" button and close your web browser.

[SUBMIT]

[EXIT PAGE]

If ins_email1 and ins_email2 NE ' ', show exit_email

exit_email

Thank you for completing the survey! The Visa electronic gift card code was just sent from [NORC EMAIL ADDRESS] to the email you provided. Please check your spam folder if you do not see it in your inbox in the next several minutes.

The information you provided will be used to help researchers and policymakers understand how MAR and OUD services can be improved throughout Illinois. If you have questions about this survey, please contact NORC by calling toll free at C or sending an email to XXXXX@norc.org.

If no_email = 1, show exit_noemail

[CIW PAGE]

exit_noemail
Thank you for completing the survey! Your Visa electronic gift card code is listed below. Please carefully write it down in a safe place so you can use it to purchase something in the future.

Visa gift card code: [XXXXXXX-XXXXX]

The information you provided will be used to help researchers and policymakers understand how MAR and OUD services can be improved throughout Illinois. If you have questions about this survey, please contact NORC by calling toll free at XXX-XXX-XXXX or sending an email to XXXXX @norc.org.

AIMS Press

© 2025 the Author(s), licensee AIMS Press. This is an open access article distributed under the terms of the Creative Commons Attribution License (https://creativecommons.org/licenses/by/4.0)
